# 1,2,4,5-Tetra­phenyl-1*H*-imidazole

**DOI:** 10.1107/S1600536812003145

**Published:** 2012-01-31

**Authors:** Bing Zhao, Zhi-yu Li, Meng-jiao Fan, Bo Song, Qi-gang Deng

**Affiliations:** a Chemistry and Chemical Engineering Institute, Qiqihar University, Heilongjiang Qiqihar 161006, People’s Republic of China

## Abstract

The asymmetric unit of the title compound, C_27_H_20_N_2_, contains two independent mol­ecules, *A* and *B*. In both mol­ecules, the N atom in the 1-position and the C atom in the 5-position are statistically disordered [as 0.571 (8):0.429 (8) in *A* and 0.736 (9):0.264 (9) in *B*]. The phenyl rings in the 1-, 2-, 4- and 5-positions in *A* are twisted from the central imidazole ring by 84.3 (2), 21.6 (2), 21.5 (2) and 75.7 (2)°, respectively. The corresponding dihedral angles in *B* are 85.5 (2), 3.8 (2), 2.4 (2) and 81.7 (2)°, respectively.

## Related literature

For the pharmacological properties of imidazole derivatives, see: Hori *et al.* (2000 ▶); Mamolo *et al.* (2004 ▶); Khabnadideh *et al.* (2003 ▶). For the crystal structure of related 2-(4-fluoro­phen­yl)-1,4,5-triphenyl-1H-imidazole, see: Gayathri *et al.* (2010 ▶).
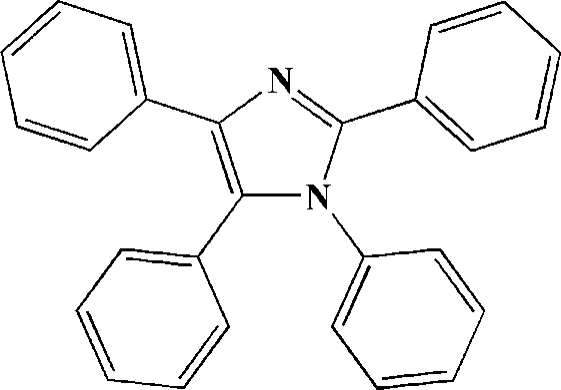


## Experimental

### 

#### Crystal data

C_27_H_20_N_2_
*M* *_r_* = 372.45
Triclinic, 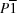

*a* = 9.8169 (15) Å
*b* = 9.8846 (15) Å
*c* = 20.601 (3) Å
α = 81.133 (5)°
β = 82.922 (6)°
γ = 84.085 (6)°
*V* = 1952.9 (5) Å^3^
*Z* = 4
Mo *K*α radiation
μ = 0.07 mm^−1^
*T* = 113 K
0.20 × 0.18 × 0.10 mm

#### Data collection

Rigaku Saturn CCD area-detector diffractometer
Absorption correction: multi-scan (*CrystalClear*; Rigaku/MSC, 2005 ▶) *T*_min_ = 0.985, *T*_max_ = 0.993
25242 measured reflections
9251 independent reflections
6476 reflections with *I* > 2σ(*I*)
*R*_int_ = 0.037

#### Refinement

*R*[*F* ^2^ > 2σ(*F* ^2^)] = 0.037
*wR*(*F* ^2^) = 0.090
*S* = 0.98
9251 reflections
525 parameters
H-atom parameters constrained
Δρ_max_ = 0.18 e Å^−3^
Δρ_min_ = −0.22 e Å^−3^

### 

Data collection: *CrystalClear* (Rigaku/MSC, 2005 ▶); cell refinement: *CrystalClear*; data reduction: *CrystalClear*; program(s) used to solve structure: *SHELXS97* (Sheldrick, 2008 ▶); program(s) used to refine structure: *SHELXL97* (Sheldrick, 2008 ▶); molecular graphics: *SHELXTL* (Sheldrick, 2008 ▶); software used to prepare material for publication: *SHELXL97*.

## Supplementary Material

Crystal structure: contains datablock(s) global, I. DOI: 10.1107/S1600536812003145/cv5236sup1.cif

Structure factors: contains datablock(s) I. DOI: 10.1107/S1600536812003145/cv5236Isup2.hkl

Supplementary material file. DOI: 10.1107/S1600536812003145/cv5236Isup3.cml

Additional supplementary materials:  crystallographic information; 3D view; checkCIF report

## Figures and Tables

**Fig. 1.**
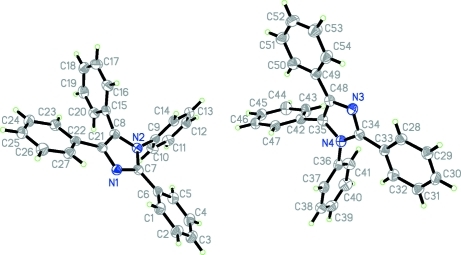
Two independent molecules in (I) showing the atom-labelling scheme. Displacement ellipsoids are drawn at the 60% probability level. For the disordered atoms, only major components are shown.

## supplementary crystallographic information

### Comment

Imidazole derivatives exhibit various pharmacological properties, such as antifungal (Hori *et al.*, 2000; Mamolo *et al.*, 2004) and antibacterial (Khabnadideh *et al.*, 2003) activities. The crystallographic structure of the similar imidazole compound had been reported . As the part of our research, the title compound 1,2,4,5-tetraphenyl-1*H*-imidazole was snythesized and its crystal structure was reported here.

The asymmetric unit of (I) contains two independent molecules (Fig. 1), *A* and *B*, respectively. All bond lengths and angles in (I) are normal and comparable with those observed in the related 2-(4-fluorophenyl)-1,4,5-triphenyl-1H-imidazole (Gayathri *et al.*, 2010). In both independent molecules, atoms N2 (N4) (1-position) and C8 (C35) (5-position) are statistically disordered in the 0.571 (8):0.429 (8) and 0.736 (9):0.264 (9) ratios, respectively, in *A* and *B*. In *A*, the imidazole ring forms the dihedral angles of 84.3 (2), 21.6 (2), 21.5 (2) and 75.7 (2)°, respectively, with the phenyl rings in the 1-, 2-, 4- and 5-positions. The corresponding dihedral angles in *B* are 85.5 (2), 3.8 (2), 2.4 (2) and 81.7 (2)°, respectively.

### Experimental

The title compound was synthesized by the reaction of the benzaldehyde (1.1 g 10 mmol), aniline (1.0 g, 10 mmol), benzil (2.1 g, 10 mmol) and ammonium acetate (4.6 g, 10 mmol) in the refluxing ethanol (20 ml) for 5 days. Crystals of (I) suitable for single-crystal X-ray analysis were grown by slow evaporation of a solution in ethanol:hexane (1:1).

### Refinement

All H atoms were positioned geometrically and refined as riding (C—H = 0.95 Å) and allowed to ride on their parent atoms, with *U*_iso_(H) = 1.2*U*_eq_ (parent).

### Crystal data

**Table:**

C_27_H_20_N_2_	*Z* = 4
*M**_r_* = 372.45	*F*(000) = 784
Triclinic, *P*1	*D*_x_ = 1.267 Mg m^−^^3^
Hall symbol: -P 1	Mo *K*α radiation, λ = 0.71073 Å
*a* = 9.8169 (15) Å	Cell parameters from 6569 reflections
*b* = 9.8846 (15) Å	θ = 2.0–27.9°
*c* = 20.601 (3) Å	µ = 0.07 mm^−^^1^
α = 81.133 (5)°	*T* = 113 K
β = 82.922 (6)°	Prism, colourless
γ = 84.085 (6)°	0.20 × 0.18 × 0.10 mm
*V* = 1952.9 (5) Å^3^	

### Data collection

**Table:**

Rigaku Saturn CCD area-detector diffractometer	9251 independent reflections
Radiation source: rotating anode	6476 reflections with *I* > 2σ(*I*)
multilayer	*R*_int_ = 0.037
Detector resolution: 14.22 pixels mm^-1^	θ_max_ = 27.9°, θ_min_ = 2.0°
φ and ω scans	*h* = −12→12
Absorption correction: multi-scan (*CrystalClear*; Rigaku/MSC, 2005)	*k* = −12→12
*T*_min_ = 0.985, *T*_max_ = 0.993	*l* = −27→27
25242 measured reflections	

### Refinement

**Table:**

Refinement on *F*^2^	Primary atom site location: structure-invariant direct methods
Least-squares matrix: full	Secondary atom site location: difference Fourier map
*R*[*F*^2^ > 2σ(*F*^2^)] = 0.037	Hydrogen site location: inferred from neighbouring sites
*wR*(*F*^2^) = 0.090	H-atom parameters constrained
*S* = 0.98	*w* = 1/[σ^2^(*F*_o_^2^) + (0.0436*P*)^2^] where *P* = (*F*_o_^2^ + 2*F*_c_^2^)/3
9251 reflections	(Δ/σ)_max_ = 0.001
525 parameters	Δρ_max_ = 0.18 e Å^−^^3^
0 restraints	Δρ_min_ = −0.22 e Å^−^^3^

### Special details

**Table:**

Geometry. All e.s.d.'s (except the e.s.d. in the dihedral angle between two l.s. planes) are estimated using the full covariance matrix. The cell e.s.d.'s are taken into account individually in the estimation of e.s.d.'s in distances, angles and torsion angles; correlations between e.s.d.'s in cell parameters are only used when they are defined by crystal symmetry. An approximate (isotropic) treatment of cell e.s.d.'s is used for estimating e.s.d.'s involving l.s. planes.
Refinement. Refinement of *F*^2^ against ALL reflections. The weighted *R*-factor *wR* and goodness of fit *S* are based on *F*^2^, conventional *R*-factors *R* are based on *F*, with *F* set to zero for negative *F*^2^. The threshold expression of *F*^2^ > σ(*F*^2^) is used only for calculating *R*-factors(gt) *etc*. and is not relevant to the choice of reflections for refinement. *R*-factors based on *F*^2^ are statistically about twice as large as those based on *F*, and *R*- factors based on ALL data will be even larger.

### Fractional atomic coordinates and isotropic or equivalent isotropic displacement parameters (Å^2^)

**Table:**

	*x*	*y*	*z*	*U*_iso_*/*U*_eq_	Occ. (<1)
N1	0.80635 (8)	1.15360 (8)	0.43441 (4)	0.02077 (19)	
N2	0.75974 (9)	0.95723 (9)	0.49889 (4)	0.0200 (2)	0.571 (8)
C8	0.82664 (9)	0.92421 (9)	0.43964 (5)	0.0209 (2)	0.571 (8)
C8'	0.75974 (9)	0.95723 (9)	0.49889 (4)	0.0200 (2)	0.429 (8)
N2'	0.82664 (9)	0.92421 (9)	0.43964 (5)	0.0209 (2)	0.429 (8)
N3	0.29316 (8)	0.79598 (8)	0.00575 (4)	0.02204 (19)	
N4	0.46590 (9)	0.70682 (9)	0.06380 (4)	0.0209 (2)	0.736 (9)
C35	0.52319 (10)	0.74836 (10)	−0.00029 (5)	0.0218 (2)	0.736 (9)
C35'	0.46590 (9)	0.70682 (9)	0.06380 (4)	0.0209 (2)	0.264 (9)
N4'	0.52319 (10)	0.74836 (10)	−0.00029 (5)	0.0218 (2)	0.264 (9)
C1	0.90696 (11)	1.20407 (11)	0.29765 (5)	0.0254 (2)	
H1	0.8430	1.2712	0.3155	0.030*	
C2	0.98019 (11)	1.23762 (12)	0.23614 (5)	0.0294 (3)	
H2	0.9666	1.3273	0.2123	0.035*	
C3	1.07316 (11)	1.14009 (12)	0.20948 (5)	0.0304 (3)	
H3	1.1244	1.1632	0.1677	0.036*	
C4	1.09098 (11)	1.00934 (12)	0.24388 (5)	0.0298 (3)	
H4	1.1533	0.9420	0.2252	0.036*	
C5	1.01853 (11)	0.97552 (11)	0.30548 (5)	0.0265 (2)	
H5	1.0320	0.8852	0.3287	0.032*	
C6	0.92601 (10)	1.07277 (11)	0.33370 (5)	0.0218 (2)	
C7	0.85347 (10)	1.04719 (10)	0.40096 (5)	0.0204 (2)	
C9	0.84989 (10)	0.78221 (10)	0.42550 (5)	0.0203 (2)	
C10	0.97511 (10)	0.70703 (11)	0.43514 (5)	0.0257 (2)	
H10	1.0459	0.7479	0.4507	0.031*	
C11	0.99713 (11)	0.57255 (11)	0.42216 (5)	0.0278 (2)	
H11	1.0835	0.5219	0.4281	0.033*	
C12	0.89327 (11)	0.51213 (11)	0.40059 (5)	0.0274 (2)	
H12	0.9079	0.4198	0.3919	0.033*	
C13	0.76796 (11)	0.58662 (11)	0.39168 (5)	0.0269 (2)	
H13	0.6966	0.5447	0.3771	0.032*	
C14	0.74547 (10)	0.72159 (11)	0.40371 (5)	0.0238 (2)	
H14	0.6595	0.7723	0.3971	0.029*	
C15	0.71475 (10)	0.85723 (10)	0.55535 (5)	0.0210 (2)	
C16	0.57519 (11)	0.84139 (11)	0.57171 (5)	0.0271 (2)	
H16	0.5102	0.8869	0.5434	0.033*	
C17	0.53132 (12)	0.75874 (12)	0.62969 (6)	0.0316 (3)	
H17	0.4360	0.7476	0.6411	0.038*	
C18	0.62606 (12)	0.69239 (11)	0.67099 (6)	0.0309 (3)	
H18	0.5956	0.6381	0.7113	0.037*	
C19	0.76538 (12)	0.70531 (11)	0.65341 (5)	0.0297 (3)	
H19	0.8305	0.6579	0.6812	0.036*	
C20	0.81003 (11)	0.78701 (11)	0.59547 (5)	0.0256 (2)	
H20	0.9057	0.7949	0.5833	0.031*	
C21	0.74965 (10)	1.09806 (10)	0.49433 (5)	0.0197 (2)	
C22	0.69161 (10)	1.18380 (10)	0.54532 (5)	0.0202 (2)	
C23	0.67869 (10)	1.13582 (11)	0.61297 (5)	0.0238 (2)	
H23	0.7025	1.0416	0.6282	0.029*	
C24	0.63125 (11)	1.22518 (11)	0.65807 (5)	0.0274 (2)	
H24	0.6218	1.1914	0.7040	0.033*	
C25	0.59758 (11)	1.36258 (11)	0.63704 (5)	0.0264 (2)	
H25	0.5658	1.4232	0.6683	0.032*	
C26	0.61036 (11)	1.41183 (11)	0.56999 (5)	0.0273 (2)	
H26	0.5879	1.5065	0.5552	0.033*	
C27	0.65588 (10)	1.32298 (10)	0.52461 (5)	0.0241 (2)	
H27	0.6630	1.3570	0.4787	0.029*	
C28	0.28857 (10)	0.91492 (10)	−0.12890 (5)	0.0235 (2)	
H28	0.2066	0.9158	−0.0991	0.028*	
C29	0.28205 (11)	0.96577 (11)	−0.19530 (5)	0.0259 (2)	
H29	0.1959	0.9999	−0.2106	0.031*	
C30	0.40074 (11)	0.96670 (11)	−0.23907 (5)	0.0266 (2)	
H30	0.3965	1.0010	−0.2845	0.032*	
C31	0.52586 (11)	0.91717 (11)	−0.21611 (5)	0.0267 (2)	
H31	0.6078	0.9189	−0.2459	0.032*	
C32	0.53262 (11)	0.86525 (11)	−0.15014 (5)	0.0255 (2)	
H32	0.6191	0.8310	−0.1352	0.031*	
C33	0.41349 (10)	0.86257 (10)	−0.10519 (5)	0.0207 (2)	
C34	0.41416 (10)	0.80371 (10)	−0.03502 (5)	0.0207 (2)	
C36	0.67340 (10)	0.73485 (10)	−0.01793 (5)	0.0220 (2)	
C37	0.75036 (11)	0.84435 (11)	−0.01528 (5)	0.0274 (2)	
H37	0.7060	0.9285	−0.0035	0.033*	
C38	0.89264 (12)	0.82932 (12)	−0.03002 (5)	0.0325 (3)	
H38	0.9453	0.9040	−0.0284	0.039*	
C39	0.95823 (11)	0.70780 (13)	−0.04689 (5)	0.0337 (3)	
H39	1.0557	0.6982	−0.0560	0.040*	
C40	0.88203 (12)	0.59959 (12)	−0.05049 (6)	0.0343 (3)	
H40	0.9268	0.5161	−0.0629	0.041*	
C41	0.74000 (11)	0.61332 (11)	−0.03586 (6)	0.0293 (3)	
H41	0.6878	0.5387	−0.0381	0.035*	
C42	0.54497 (10)	0.64411 (10)	0.11728 (5)	0.0213 (2)	
C43	0.58342 (11)	0.50406 (11)	0.12473 (5)	0.0284 (2)	
H43	0.5627	0.4502	0.0934	0.034*	
C44	0.65214 (12)	0.44297 (11)	0.17803 (5)	0.0308 (3)	
H44	0.6788	0.3471	0.1834	0.037*	
C45	0.68173 (11)	0.52238 (11)	0.22345 (5)	0.0275 (2)	
H45	0.7280	0.4806	0.2603	0.033*	
C46	0.64419 (10)	0.66234 (11)	0.21547 (5)	0.0259 (2)	
H46	0.6648	0.7162	0.2469	0.031*	
C47	0.57684 (10)	0.72411 (11)	0.16203 (5)	0.0236 (2)	
H47	0.5527	0.8204	0.1560	0.028*	
C48	0.32554 (10)	0.73797 (10)	0.06543 (5)	0.0214 (2)	
C49	0.21973 (10)	0.71348 (10)	0.12231 (5)	0.0224 (2)	
C50	0.24418 (11)	0.64767 (11)	0.18527 (5)	0.0300 (3)	
H50	0.3354	0.6141	0.1938	0.036*	
C51	0.13686 (11)	0.63061 (12)	0.23557 (6)	0.0322 (3)	
H51	0.1557	0.5873	0.2784	0.039*	
C52	0.00356 (12)	0.67575 (12)	0.22413 (5)	0.0318 (3)	
H52	−0.0697	0.6629	0.2586	0.038*	
C53	−0.02245 (12)	0.74022 (13)	0.16166 (6)	0.0355 (3)	
H53	−0.1142	0.7713	0.1531	0.043*	
C54	0.08427 (11)	0.75956 (12)	0.11180 (5)	0.0304 (3)	
H54	0.0649	0.8052	0.0695	0.037*	

### Atomic displacement parameters (Å^2^)

**Table:**

	*U*^11^	*U*^22^	*U*^33^	*U*^12^	*U*^13^	*U*^23^
N1	0.0220 (4)	0.0206 (5)	0.0197 (4)	−0.0017 (3)	−0.0024 (3)	−0.0027 (4)
N2	0.0189 (5)	0.0193 (5)	0.0218 (5)	−0.0015 (4)	−0.0022 (4)	−0.0030 (4)
C8	0.0201 (5)	0.0198 (5)	0.0230 (5)	−0.0011 (4)	−0.0028 (4)	−0.0035 (4)
C8'	0.0189 (5)	0.0193 (5)	0.0218 (5)	−0.0015 (4)	−0.0022 (4)	−0.0030 (4)
N2'	0.0201 (5)	0.0198 (5)	0.0230 (5)	−0.0011 (4)	−0.0028 (4)	−0.0035 (4)
N3	0.0240 (5)	0.0212 (5)	0.0203 (4)	0.0004 (4)	−0.0027 (4)	−0.0024 (4)
N4	0.0224 (5)	0.0193 (5)	0.0208 (5)	0.0005 (4)	−0.0037 (4)	−0.0029 (4)
C35	0.0237 (5)	0.0201 (5)	0.0218 (5)	0.0006 (4)	−0.0037 (4)	−0.0042 (4)
C35'	0.0224 (5)	0.0193 (5)	0.0208 (5)	0.0005 (4)	−0.0037 (4)	−0.0029 (4)
N4'	0.0237 (5)	0.0201 (5)	0.0218 (5)	0.0006 (4)	−0.0037 (4)	−0.0042 (4)
C1	0.0253 (6)	0.0275 (6)	0.0231 (5)	0.0008 (4)	−0.0038 (4)	−0.0038 (4)
C2	0.0293 (6)	0.0335 (6)	0.0239 (6)	−0.0019 (5)	−0.0049 (5)	0.0016 (5)
C3	0.0255 (6)	0.0440 (7)	0.0210 (5)	−0.0031 (5)	−0.0027 (5)	−0.0022 (5)
C4	0.0241 (6)	0.0383 (7)	0.0270 (6)	0.0024 (5)	−0.0013 (5)	−0.0093 (5)
C5	0.0258 (6)	0.0273 (6)	0.0263 (6)	0.0000 (5)	−0.0037 (5)	−0.0050 (5)
C6	0.0206 (5)	0.0252 (6)	0.0208 (5)	−0.0030 (4)	−0.0046 (4)	−0.0049 (4)
C7	0.0193 (5)	0.0200 (5)	0.0226 (5)	−0.0020 (4)	−0.0042 (4)	−0.0033 (4)
C9	0.0222 (5)	0.0200 (5)	0.0177 (5)	−0.0023 (4)	−0.0003 (4)	−0.0007 (4)
C10	0.0218 (5)	0.0234 (6)	0.0314 (6)	−0.0032 (4)	−0.0041 (4)	−0.0011 (5)
C11	0.0253 (6)	0.0245 (6)	0.0307 (6)	0.0034 (5)	−0.0011 (5)	−0.0001 (5)
C12	0.0360 (6)	0.0213 (6)	0.0242 (6)	−0.0015 (5)	0.0014 (5)	−0.0053 (5)
C13	0.0292 (6)	0.0292 (6)	0.0243 (6)	−0.0068 (5)	−0.0033 (5)	−0.0072 (5)
C14	0.0219 (5)	0.0278 (6)	0.0216 (5)	−0.0009 (4)	−0.0028 (4)	−0.0037 (4)
C15	0.0228 (5)	0.0190 (5)	0.0225 (5)	−0.0023 (4)	−0.0013 (4)	−0.0072 (4)
C16	0.0236 (6)	0.0259 (6)	0.0331 (6)	−0.0023 (5)	−0.0040 (5)	−0.0074 (5)
C17	0.0268 (6)	0.0316 (6)	0.0370 (7)	−0.0084 (5)	0.0050 (5)	−0.0098 (5)
C18	0.0425 (7)	0.0226 (6)	0.0276 (6)	−0.0071 (5)	0.0033 (5)	−0.0067 (5)
C19	0.0367 (7)	0.0235 (6)	0.0297 (6)	−0.0001 (5)	−0.0087 (5)	−0.0045 (5)
C20	0.0227 (6)	0.0244 (6)	0.0304 (6)	−0.0009 (4)	−0.0030 (5)	−0.0070 (5)
C21	0.0179 (5)	0.0193 (5)	0.0222 (5)	−0.0014 (4)	−0.0044 (4)	−0.0026 (4)
C22	0.0167 (5)	0.0215 (5)	0.0228 (5)	−0.0033 (4)	−0.0018 (4)	−0.0035 (4)
C23	0.0271 (6)	0.0211 (5)	0.0229 (5)	−0.0028 (4)	−0.0044 (4)	−0.0006 (4)
C24	0.0341 (6)	0.0281 (6)	0.0201 (5)	−0.0055 (5)	−0.0013 (5)	−0.0034 (5)
C25	0.0269 (6)	0.0259 (6)	0.0274 (6)	−0.0030 (5)	0.0016 (5)	−0.0100 (5)
C26	0.0290 (6)	0.0209 (6)	0.0308 (6)	0.0012 (5)	−0.0002 (5)	−0.0044 (5)
C27	0.0264 (6)	0.0220 (6)	0.0223 (5)	−0.0003 (4)	−0.0004 (4)	−0.0011 (4)
C28	0.0223 (5)	0.0244 (6)	0.0233 (5)	−0.0016 (4)	−0.0013 (4)	−0.0030 (4)
C29	0.0275 (6)	0.0246 (6)	0.0264 (6)	−0.0010 (4)	−0.0078 (5)	−0.0029 (5)
C30	0.0360 (6)	0.0251 (6)	0.0189 (5)	−0.0039 (5)	−0.0046 (5)	−0.0022 (4)
C31	0.0279 (6)	0.0285 (6)	0.0231 (5)	−0.0027 (5)	0.0025 (4)	−0.0055 (5)
C32	0.0240 (6)	0.0271 (6)	0.0250 (5)	0.0012 (4)	−0.0025 (4)	−0.0051 (5)
C33	0.0244 (5)	0.0178 (5)	0.0204 (5)	−0.0014 (4)	−0.0031 (4)	−0.0043 (4)
C34	0.0217 (5)	0.0181 (5)	0.0230 (5)	0.0006 (4)	−0.0026 (4)	−0.0059 (4)
C36	0.0246 (5)	0.0236 (6)	0.0177 (5)	0.0001 (4)	−0.0053 (4)	−0.0014 (4)
C37	0.0347 (6)	0.0267 (6)	0.0216 (5)	−0.0040 (5)	−0.0042 (5)	−0.0038 (5)
C38	0.0334 (6)	0.0393 (7)	0.0262 (6)	−0.0141 (5)	−0.0060 (5)	0.0003 (5)
C39	0.0239 (6)	0.0457 (8)	0.0280 (6)	−0.0015 (5)	−0.0028 (5)	0.0048 (5)
C40	0.0301 (6)	0.0326 (7)	0.0372 (7)	0.0057 (5)	−0.0022 (5)	−0.0025 (5)
C41	0.0268 (6)	0.0249 (6)	0.0358 (6)	−0.0008 (5)	−0.0036 (5)	−0.0044 (5)
C42	0.0197 (5)	0.0227 (5)	0.0209 (5)	−0.0005 (4)	−0.0021 (4)	−0.0026 (4)
C43	0.0359 (6)	0.0235 (6)	0.0277 (6)	−0.0002 (5)	−0.0084 (5)	−0.0074 (5)
C44	0.0383 (7)	0.0215 (6)	0.0320 (6)	0.0037 (5)	−0.0088 (5)	−0.0021 (5)
C45	0.0286 (6)	0.0299 (6)	0.0229 (5)	0.0002 (5)	−0.0056 (5)	0.0001 (5)
C46	0.0269 (6)	0.0293 (6)	0.0227 (5)	−0.0033 (5)	−0.0032 (4)	−0.0071 (5)
C47	0.0252 (6)	0.0218 (5)	0.0235 (5)	−0.0008 (4)	−0.0011 (4)	−0.0045 (4)
C48	0.0227 (5)	0.0193 (5)	0.0223 (5)	0.0008 (4)	−0.0045 (4)	−0.0041 (4)
C49	0.0252 (6)	0.0203 (5)	0.0219 (5)	−0.0020 (4)	−0.0022 (4)	−0.0039 (4)
C50	0.0262 (6)	0.0331 (6)	0.0279 (6)	−0.0009 (5)	−0.0035 (5)	0.0039 (5)
C51	0.0341 (7)	0.0344 (7)	0.0254 (6)	−0.0049 (5)	−0.0023 (5)	0.0047 (5)
C52	0.0291 (6)	0.0372 (7)	0.0268 (6)	−0.0048 (5)	0.0035 (5)	−0.0016 (5)
C53	0.0244 (6)	0.0491 (8)	0.0304 (6)	0.0021 (5)	−0.0016 (5)	−0.0025 (6)
C54	0.0279 (6)	0.0396 (7)	0.0221 (5)	0.0022 (5)	−0.0041 (5)	−0.0015 (5)

### Geometric parameters (Å, °)

**Table:**

N1—C21	1.3490 (12)	C24—C25	1.3794 (15)
N1—C7	1.3563 (13)	C24—H24	0.9500
N2—C21	1.3748 (13)	C25—C26	1.3870 (15)
N2—C8	1.3815 (12)	C25—H25	0.9500
N2—C15	1.4607 (13)	C26—C27	1.3836 (14)
C8—C7	1.3775 (13)	C26—H26	0.9500
C8—C9	1.4666 (13)	C27—H27	0.9500
N3—C48	1.3353 (12)	C28—C29	1.3895 (14)
N3—C34	1.3694 (13)	C28—C33	1.3961 (14)
N4—C48	1.3779 (13)	C28—H28	0.9500
N4—C35	1.3875 (12)	C29—C30	1.3826 (15)
N4—C42	1.4498 (13)	C29—H29	0.9500
C35—C34	1.3800 (13)	C30—C31	1.3857 (15)
C35—C36	1.4719 (14)	C30—H30	0.9500
C1—C2	1.3883 (14)	C31—C32	1.3839 (14)
C1—C6	1.3992 (14)	C31—H31	0.9500
C1—H1	0.9500	C32—C33	1.3993 (14)
C2—C3	1.3865 (15)	C32—H32	0.9500
C2—H2	0.9500	C33—C34	1.4732 (14)
C3—C4	1.3802 (15)	C36—C41	1.3866 (14)
C3—H3	0.9500	C36—C37	1.3936 (14)
C4—C5	1.3869 (15)	C37—C38	1.3899 (15)
C4—H4	0.9500	C37—H37	0.9500
C5—C6	1.3962 (14)	C38—C39	1.3756 (16)
C5—H5	0.9500	C38—H38	0.9500
C6—C7	1.4756 (14)	C39—C40	1.3828 (16)
C9—C10	1.3890 (14)	C39—H39	0.9500
C9—C14	1.3907 (14)	C40—C41	1.3868 (15)
C10—C11	1.3861 (14)	C40—H40	0.9500
C10—H10	0.9500	C41—H41	0.9500
C11—C12	1.3830 (15)	C42—C47	1.3856 (14)
C11—H11	0.9500	C42—C43	1.3869 (14)
C12—C13	1.3844 (15)	C43—C44	1.3854 (15)
C12—H12	0.9500	C43—H43	0.9500
C13—C14	1.3848 (14)	C44—C45	1.3845 (15)
C13—H13	0.9500	C44—H44	0.9500
C14—H14	0.9500	C45—C46	1.3844 (15)
C15—C20	1.3856 (14)	C45—H45	0.9500
C15—C16	1.3890 (14)	C46—C47	1.3823 (14)
C16—C17	1.3872 (15)	C46—H46	0.9500
C16—H16	0.9500	C47—H47	0.9500
C17—C18	1.3843 (16)	C48—C49	1.4748 (14)
C17—H17	0.9500	C49—C54	1.3922 (14)
C18—C19	1.3848 (15)	C49—C50	1.3956 (14)
C18—H18	0.9500	C50—C51	1.3878 (14)
C19—C20	1.3841 (15)	C50—H50	0.9500
C19—H19	0.9500	C51—C52	1.3749 (15)
C20—H20	0.9500	C51—H51	0.9500
C21—C22	1.4744 (14)	C52—C53	1.3865 (15)
C22—C23	1.3959 (14)	C52—H52	0.9500
C22—C27	1.3979 (14)	C53—C54	1.3801 (15)
C23—C24	1.3863 (14)	C53—H53	0.9500
C23—H23	0.9500	C54—H54	0.9500
			
C21—N1—C7	106.53 (8)	C26—C25—H25	120.2
C21—N2—C8	107.05 (8)	C27—C26—C25	119.92 (10)
C21—N2—C15	128.13 (8)	C27—C26—H26	120.0
C8—N2—C15	124.78 (8)	C25—C26—H26	120.0
C7—C8—N2	106.16 (8)	C26—C27—C22	120.95 (10)
C7—C8—C9	131.46 (9)	C26—C27—H27	119.5
N2—C8—C9	122.27 (8)	C22—C27—H27	119.5
C48—N3—C34	106.87 (8)	C29—C28—C33	121.15 (10)
C48—N4—C35	107.27 (8)	C29—C28—H28	119.4
C48—N4—C42	128.57 (9)	C33—C28—H28	119.4
C35—N4—C42	124.16 (8)	C30—C29—C28	120.08 (10)
C34—C35—N4	105.81 (9)	C30—C29—H29	120.0
C34—C35—C36	133.24 (9)	C28—C29—H29	120.0
N4—C35—C36	120.88 (9)	C29—C30—C31	119.47 (10)
C2—C1—C6	120.93 (10)	C29—C30—H30	120.3
C2—C1—H1	119.5	C31—C30—H30	120.3
C6—C1—H1	119.5	C32—C31—C30	120.62 (10)
C3—C2—C1	119.96 (11)	C32—C31—H31	119.7
C3—C2—H2	120.0	C30—C31—H31	119.7
C1—C2—H2	120.0	C31—C32—C33	120.72 (10)
C4—C3—C2	119.75 (10)	C31—C32—H32	119.6
C4—C3—H3	120.1	C33—C32—H32	119.6
C2—C3—H3	120.1	C28—C33—C32	117.94 (10)
C3—C4—C5	120.50 (10)	C28—C33—C34	119.13 (9)
C3—C4—H4	119.7	C32—C33—C34	122.91 (9)
C5—C4—H4	119.7	N3—C34—C35	109.90 (9)
C4—C5—C6	120.68 (10)	N3—C34—C33	120.32 (9)
C4—C5—H5	119.7	C35—C34—C33	129.77 (9)
C6—C5—H5	119.7	C41—C36—C37	119.41 (10)
C5—C6—C1	118.16 (10)	C41—C36—C35	120.82 (9)
C5—C6—C7	123.56 (10)	C37—C36—C35	119.76 (9)
C1—C6—C7	118.18 (9)	C38—C37—C36	119.42 (10)
N1—C7—C8	110.23 (9)	C38—C37—H37	120.3
N1—C7—C6	120.35 (9)	C36—C37—H37	120.3
C8—C7—C6	129.38 (9)	C39—C38—C37	120.87 (10)
C10—C9—C14	119.85 (10)	C39—C38—H38	119.6
C10—C9—C8	120.13 (9)	C37—C38—H38	119.6
C14—C9—C8	120.02 (9)	C38—C39—C40	119.88 (11)
C11—C10—C9	120.27 (10)	C38—C39—H39	120.1
C11—C10—H10	119.9	C40—C39—H39	120.1
C9—C10—H10	119.9	C39—C40—C41	119.78 (11)
C12—C11—C10	119.91 (10)	C39—C40—H40	120.1
C12—C11—H11	120.0	C41—C40—H40	120.1
C10—C11—H11	120.0	C36—C41—C40	120.64 (10)
C11—C12—C13	119.79 (10)	C36—C41—H41	119.7
C11—C12—H12	120.1	C40—C41—H41	119.7
C13—C12—H12	120.1	C47—C42—C43	120.60 (10)
C12—C13—C14	120.75 (10)	C47—C42—N4	119.48 (9)
C12—C13—H13	119.6	C43—C42—N4	119.88 (9)
C14—C13—H13	119.6	C44—C43—C42	119.79 (9)
C13—C14—C9	119.42 (10)	C44—C43—H43	120.1
C13—C14—H14	120.3	C42—C43—H43	120.1
C9—C14—H14	120.3	C45—C44—C43	119.65 (10)
C20—C15—C16	120.19 (10)	C45—C44—H44	120.2
C20—C15—N2	119.91 (9)	C43—C44—H44	120.2
C16—C15—N2	119.68 (9)	C46—C45—C44	120.33 (10)
C17—C16—C15	119.61 (10)	C46—C45—H45	119.8
C17—C16—H16	120.2	C44—C45—H45	119.8
C15—C16—H16	120.2	C47—C46—C45	120.29 (10)
C18—C17—C16	120.21 (10)	C47—C46—H46	119.9
C18—C17—H17	119.9	C45—C46—H46	119.9
C16—C17—H17	119.9	C46—C47—C42	119.33 (10)
C17—C18—C19	119.88 (11)	C46—C47—H47	120.3
C17—C18—H18	120.1	C42—C47—H47	120.3
C19—C18—H18	120.1	N3—C48—N4	110.15 (9)
C20—C19—C18	120.24 (11)	N3—C48—C49	121.94 (9)
C20—C19—H19	119.9	N4—C48—C49	127.91 (9)
C18—C19—H19	119.9	C54—C49—C50	117.67 (10)
C19—C20—C15	119.79 (10)	C54—C49—C48	116.86 (9)
C19—C20—H20	120.1	C50—C49—C48	125.47 (10)
C15—C20—H20	120.1	C51—C50—C49	120.81 (10)
N1—C21—N2	110.03 (8)	C51—C50—H50	119.6
N1—C21—C22	121.72 (9)	C49—C50—H50	119.6
N2—C21—C22	128.22 (9)	C52—C51—C50	120.71 (11)
C23—C22—C27	118.47 (9)	C52—C51—H51	119.6
C23—C22—C21	123.71 (9)	C50—C51—H51	119.6
C27—C22—C21	117.69 (9)	C51—C52—C53	119.11 (10)
C24—C23—C22	120.21 (10)	C51—C52—H52	120.4
C24—C23—H23	119.9	C53—C52—H52	120.4
C22—C23—H23	119.9	C54—C53—C52	120.37 (11)
C25—C24—C23	120.77 (10)	C54—C53—H53	119.8
C25—C24—H24	119.6	C52—C53—H53	119.8
C23—C24—H24	119.6	C53—C54—C49	121.32 (10)
C24—C25—C26	119.67 (10)	C53—C54—H54	119.3
C24—C25—H25	120.2	C49—C54—H54	119.3
			
C21—N2—C8—C7	−0.59 (11)	C24—C25—C26—C27	0.41 (16)
C15—N2—C8—C7	−178.50 (9)	C25—C26—C27—C22	−1.04 (16)
C21—N2—C8—C9	−177.05 (8)	C23—C22—C27—C26	0.80 (15)
C15—N2—C8—C9	5.04 (14)	C21—C22—C27—C26	−175.23 (9)
C48—N4—C35—C34	0.15 (10)	C33—C28—C29—C30	−0.78 (15)
C42—N4—C35—C34	−179.23 (8)	C28—C29—C30—C31	−0.29 (15)
C48—N4—C35—C36	177.31 (9)	C29—C30—C31—C32	0.90 (16)
C42—N4—C35—C36	−2.07 (14)	C30—C31—C32—C33	−0.46 (16)
C6—C1—C2—C3	0.37 (16)	C29—C28—C33—C32	1.20 (15)
C1—C2—C3—C4	0.93 (16)	C29—C28—C33—C34	−177.08 (9)
C2—C3—C4—C5	−1.23 (16)	C31—C32—C33—C28	−0.58 (15)
C3—C4—C5—C6	0.23 (16)	C31—C32—C33—C34	177.62 (9)
C4—C5—C6—C1	1.04 (15)	C48—N3—C34—C35	0.63 (11)
C4—C5—C6—C7	−175.23 (10)	C48—N3—C34—C33	179.57 (8)
C2—C1—C6—C5	−1.34 (15)	N4—C35—C34—N3	−0.48 (11)
C2—C1—C6—C7	175.14 (9)	C36—C35—C34—N3	−177.14 (10)
C21—N1—C7—C8	0.01 (11)	N4—C35—C34—C33	−179.29 (9)
C21—N1—C7—C6	−178.04 (9)	C36—C35—C34—C33	4.06 (18)
N2—C8—C7—N1	0.36 (11)	C28—C33—C34—N3	2.37 (14)
C9—C8—C7—N1	176.37 (10)	C32—C33—C34—N3	−175.81 (9)
N2—C8—C7—C6	178.19 (10)	C28—C33—C34—C35	−178.93 (10)
C9—C8—C7—C6	−5.80 (18)	C32—C33—C34—C35	2.88 (16)
C5—C6—C7—N1	156.07 (9)	C34—C35—C36—C41	−97.65 (14)
C1—C6—C7—N1	−20.20 (14)	N4—C35—C36—C41	86.10 (12)
C5—C6—C7—C8	−21.57 (16)	C34—C35—C36—C37	83.72 (14)
C1—C6—C7—C8	162.16 (10)	N4—C35—C36—C37	−92.53 (12)
C7—C8—C9—C10	87.87 (14)	C41—C36—C37—C38	−0.65 (15)
N2—C8—C9—C10	−96.66 (12)	C35—C36—C37—C38	178.00 (9)
C7—C8—C9—C14	−93.46 (13)	C36—C37—C38—C39	−0.25 (16)
N2—C8—C9—C14	82.00 (12)	C37—C38—C39—C40	1.19 (17)
C14—C9—C10—C11	0.98 (15)	C38—C39—C40—C41	−1.21 (17)
C8—C9—C10—C11	179.65 (9)	C37—C36—C41—C40	0.62 (16)
C9—C10—C11—C12	−1.08 (16)	C35—C36—C41—C40	−178.02 (10)
C10—C11—C12—C13	0.38 (16)	C39—C40—C41—C36	0.31 (17)
C11—C12—C13—C14	0.43 (16)	C48—N4—C42—C47	−80.28 (13)
C12—C13—C14—C9	−0.53 (15)	C35—N4—C42—C47	98.96 (11)
C10—C9—C14—C13	−0.17 (15)	C48—N4—C42—C43	97.24 (12)
C8—C9—C14—C13	−178.84 (9)	C35—N4—C42—C43	−83.52 (12)
C21—N2—C15—C20	−100.75 (12)	C47—C42—C43—C44	1.16 (16)
C8—N2—C15—C20	76.71 (12)	N4—C42—C43—C44	−176.33 (9)
C21—N2—C15—C16	73.93 (13)	C42—C43—C44—C45	0.07 (17)
C8—N2—C15—C16	−108.61 (11)	C43—C44—C45—C46	−0.63 (16)
C20—C15—C16—C17	2.31 (15)	C44—C45—C46—C47	−0.03 (16)
N2—C15—C16—C17	−172.35 (9)	C45—C46—C47—C42	1.24 (15)
C15—C16—C17—C18	−0.01 (15)	C43—C42—C47—C46	−1.81 (15)
C16—C17—C18—C19	−1.93 (16)	N4—C42—C47—C46	175.69 (9)
C17—C18—C19—C20	1.59 (16)	C34—N3—C48—N4	−0.53 (11)
C18—C19—C20—C15	0.70 (15)	C34—N3—C48—C49	179.85 (9)
C16—C15—C20—C19	−2.65 (15)	C35—N4—C48—N3	0.24 (11)
N2—C15—C20—C19	172.00 (9)	C42—N4—C48—N3	179.58 (9)
C7—N1—C21—N2	−0.39 (11)	C35—N4—C48—C49	179.83 (9)
C7—N1—C21—C22	177.82 (9)	C42—N4—C48—C49	−0.83 (16)
C8—N2—C21—N1	0.62 (11)	N3—C48—C49—C54	−2.92 (14)
C15—N2—C21—N1	178.44 (9)	N4—C48—C49—C54	177.54 (10)
C8—N2—C21—C22	−177.45 (9)	N3—C48—C49—C50	176.73 (10)
C15—N2—C21—C22	0.37 (16)	N4—C48—C49—C50	−2.81 (17)
N1—C21—C22—C23	−155.58 (10)	C54—C49—C50—C51	−0.73 (16)
N2—C21—C22—C23	22.29 (16)	C48—C49—C50—C51	179.62 (10)
N1—C21—C22—C27	20.22 (14)	C49—C50—C51—C52	1.32 (17)
N2—C21—C22—C27	−161.91 (10)	C50—C51—C52—C53	−0.72 (17)
C27—C22—C23—C24	0.07 (15)	C51—C52—C53—C54	−0.42 (17)
C21—C22—C23—C24	175.83 (9)	C52—C53—C54—C49	1.01 (17)
C22—C23—C24—C25	−0.69 (16)	C50—C49—C54—C53	−0.42 (16)
C23—C24—C25—C26	0.45 (16)	C48—C49—C54—C53	179.26 (10)
